# Considering What We Know and What We Don’t Know: Expectations and Confidence Guide Value Integration in Value-Based Decision-Making

**DOI:** 10.1162/opmi.a.3

**Published:** 2025-06-25

**Authors:** Romy Frömer, Frederick Callaway, Thomas L. Griffiths, Amitai Shenhav

**Affiliations:** Cognitive, Linguistic and Psychological Sciences, Brown University, Providence, RI, USA; School of Psychology, Centre for Human Brain Health, University of Birmingham, Birmingham, UK; Department of Psychology, Princeton University, Princeton, NJ; Department of Psychology, New York University, New York, NY, USA; Department of Psychology, University of California, Berkeley, Berkeley, CA, USA; Helen Wills Neuroscience Institute, University of California, Berkeley, Berkeley, CA, USA

**Keywords:** decision-making, metacognition, Bayesian inference, attention, sequential sampling

## Abstract

When making decisions, we often have more information about some options than others. Previous work has shown that people are more likely to choose options that they look at more and those that they are more confident in. But should one always prefer options one knows more about? Intuition suggests not. Rather, how additional information impacts our preferences should depend critically on how valuable we expect the options to be. Here, we formalize this intuition in a Bayesian sequential sampling model where attention and confidence influence the precision of momentary evidence. Our model makes a key prediction: attention and confidence both increase choice probability for better-than-average options, and both decrease choice probability for worse-than-average options. We confirm this prediction in two experiments in which we independently manipulate value and attention. Our results offer a novel perspective on prior work on the role of attention and confidence in decision-making, showing that people rely on contextual knowledge and uncertainty estimates to adaptively learn about their options and make better decisions.

## INTRODUCTION

There you are, at the overrun ice cream parlor. It is finally your turn, but despite having glared at the options for the last 10 minutes while waiting in line, you have not decided. The Stracciatella caught your eye early on, and you kept looking at it, imagining its creamy texture on your tongue occasionally interrupted by crispy chocolate chips. But as you moved down the line and caught glimpses of new flavors, you came to find it much less exciting than the pistachio blackberry swirl you just came to discover. You have never had that. Should you change your mind now? The Stracciatella would be good for sure, but the pistachio blackberry swirl might be amazing! Or, it could be a huge disappointment and make you sob for that Stracciatella.

This example illustrates two ways in which decision-making is challenged by uncertainty. On one hand, information about our options becomes available asynchronously, for instance when we sequentially attend to our options. On the other hand, the quality of available information varies between options, for instance because we are more confident in the value of one compared to the other. How do decision-makers overcome these challenges?

Previous research has characterized the downstream consequences of limited and imbalanced information on choice, and formalized the mechanisms underlying these consequences in computational models. In particular, previous work has shown that people are less likely to choose options that they look at less (Armel et al., 2008; Glickman et al., 2018; Shimojo et al., 2003; Usher et al., 2019) as well as items whose value they are less confident about (Lee & Coricelli, 2020; Polanía et al., 2019; Quandt et al., 2022). To explain these findings, attention and value confidence have each been proposed to affect the rate at which evidence in favor of an option is accumulated (Krajbich et al., 2010; Lee & Usher, 2023). However, these models assume that people passively accumulate distorted evidence. Therefore, it remains unclear what adaptive mechanisms people may use to actively compensate for limited and imbalanced information about their choice options.

Here, we address this gap, asking how people could flexibly and dynamically adapt to the challenges limited and imbalanced information pose for decision-making. We propose that people compensate for missing or imprecise information by drawing on expectations or contextual knowledge about likely option values (cf. Khaw et al., 2017; Polanía et al., 2019). Formally, we propose a Bayesian extension of classic sequential sampling models (Callaway et al., 2021; Drugowitsch et al., 2012; Jang et al., 2021; Moreno-Bote, 2010; Tajima et al., 2016) in which attention and value confidence affect the quantity and quality of information about each item’s value, which is integrated with a prior distribution to form posterior value estimates for each item.

**Figure 1.**
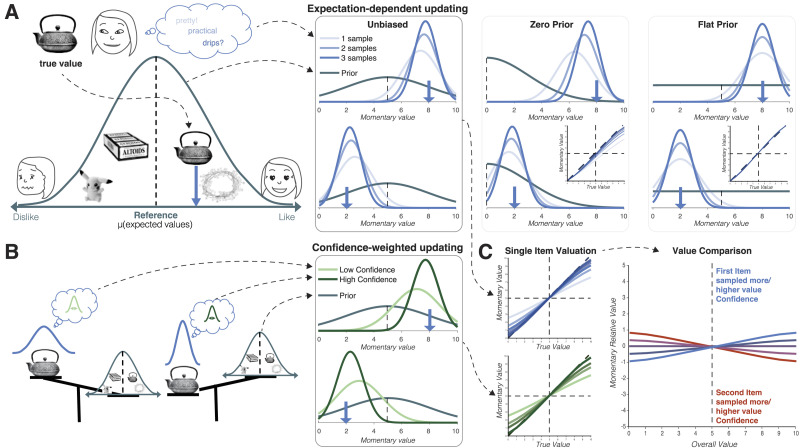
**Decision-making as sequential Bayesian updating predicts reference-dependent effects of attention and value confidence on choice.** (A) Prior expectations about the distribution of values in a given context (teal curve) influence how true item values (blue arrow) are inferred. Increasing amounts of information (increasingly blue curves) refine value estimates from the initial prior estimate towards the true value (blue arrow). With an unbiased prior, sampling typically increases the estimated value of better-than-average items (top), but decreases the estimate value for worse-than-average items (bottom). If, instead, the prior mean is zero (the minimum item value), sampling uniformly increases the momentary value estimate for all options. Alternatively, without expectations (flat prior), the estimated value will, on average, be exactly the true value, regardless of how much information is available. (B) Value confidence reflects the precision of the value representation underlying sampling. It thus determines the balance between samples and prior. When value confidence is high, samples are given greater weight and momentary value estimates are updated more. Similar to increasing the number of samples (e.g., through attention to an item), higher confidence leads to greater updating of the momentary value estimate away from the prior. Thus, like attention, value confidence has opposite effects for values above (top) and below (bottom) the mean. (C) Attention-guided sampling and value confidence similarly affect biases in momentary value estimates. Left panels show how momentary estimates systematically deviate from true values as a function of sampling and value confidence. Both decrease biases induced by the prior, hence for the representative (unbiased) prior highlighted in A and B, biases towards the average decrease with increased sampling and higher value confidence. During value comparison, asymmetries in sampling and value confidence induce systematic differences in momentary value estimates that translate into value-dependent choice biases. Increased sampling and higher confidence for one option over the other increase its choice probability when values are above the mean, but decrease it when values are below the mean.

As illustrated in Figure 1, our model makes the striking prediction that both confidence and attention should have opposite effects on choice probability for items that are better vs. worse than average. Items that are attended longer or for which value confidence is higher should be more likely to be chosen if their value is above the mean value, but less likely to be chosen if it is below the mean. We test and confirm this prediction in two experiments using a paradigm that allows us to manipulate attention independently of value. Taken together our results support the idea that decision-makers dynamically cope with uncertainty by considering both what they know and what they don’t know.

## RESULTS

### Bayesian Evidence Accumulation Model

Following previous work (Callaway et al., 2021; Fudenberg et al., 2018; Jang et al., 2021; Tajima et al., 2016, 2019), we model value-based decision-making as an iterative process in which an agent forms posterior estimates of the value of each choice option based on a sequence of noisy value samples generated by mental simulation and/or memory recall (Shadlen & Shohamy, 2016). Like most sequential sampling models of value-based choice, this class of models assumes that the decision is generated by a sequence of Gaussian samples whose mean depends on the value of each option in the choice set. However, unlike dominant sequential sampling models of value based choice (e.g., drift diffusion models and variants; Krajbich et al., 2010; Lee & Usher, 2023; Pisauro et al., 2017; Polanía et al., 2014; Sepulveda et al., 2020), which assume that the value samples are accumulated by simple addition, this newer class of models instead assumes that the samples are accumulated optimally, by Bayesian inference.

Concretely, the agent estimates the true value of each item by inferring a posterior over the mean of the distribution from which the samples are drawn. The agent continues sampling as long as it expects the benefit of doing so to exceed the cost. Once the expected improvement in the decision no longer justifies the cost associated with sampling, the agent chooses the option that currently has the highest estimated value.

On a given trial, the agent is presented with *n* items, each of which is associated with some utility, $u_{\text{true}}^{\left(i\right)}$. The agent’s goal is to select the item with maximal utility, but the agent does not have direct access to these values. Instead, they must estimate the utilities by sampling. At each time step, the agent attends to one of the available options, *i*, and receives a noisy signal of its subjective value,

$$x_{t}∼\text{Normal}\left(u_{\text{true}}^{\left(i\right)},\frac{1}{\tau^{\left(i\right)}}\right).$$ (1)

Here, *τ*^(*i*)^ is the precision (inverse variance) of the samples, capturing differences in underlying confidence about the value of each option (see Methods for details).

Given a sequence of samples and a prior Normal(*μ*_prior_, 1λprior), the agent forms a posterior distribution over each item’s value, Normal(μti,1λti). Here, $\mu_{t}^{\left(i\right)}$ is the posterior mean (the estimated value) and $\lambda_{t}^{\left(i\right)}$ is the posterior precision (the certainty in that estimate). The posterior can be expressed in terms of the total number of samples taken for the item so far, $N_{t}^{\left(i\right)}$, and the mean of those samples, ${\bar{x}}_{t}^{\left(i\right)}$:

$$\begin{array}{l} \mu_{t}^{\left(i\right)}=\mu_{\text{prior}}+\frac{N_{t}^{\left(i\right)}\tau_{t}^{\left(i\right)}}{\lambda_{t}^{\left(i\right)}}\left({\bar{x}}_{t}^{\left(i\right)}-\mu_{\text{prior}}\right)\\ \lambda_{t}^{\left(i\right)}=\lambda_{\text{prior}}+N_{t}^{\left(i\right)}\tau^{\left(i\right)} \end{array}$$ (2)

We have written the posterior in this way to emphasize two key properties of Bayesian updating. First, the impact of the samples on estimated value does not depend on their values *per se*, but rather on their values relative to one’s expectations. In particular, the posterior mean begins at the prior mean (*μ*_prior_) and is shifted up or down depending on whether the sample mean ($\bar{x}$) is greater or less than the prior mean. This property, *expectation-dependent updating*, is illustrated in Figure 1A. Second, the degree to which the estimated value is shifted towards the sample mean (and, in expectation, the true value) depends on the precision of available information. This precision is the product of the quantity (number of samples, $N_{t}^{\left(i\right)}$) and quality (value confidence, *τ*^(*i*)^) of sampled information. The greater the precision, the larger the shift towards the sample mean—or, conversely, the weaker the bias towards the prior mean. This property, *precision-weighted updating* is illustrated in Figure 1B.

The most striking prediction of this model results from the interaction of these two properties, expectation dependence and precision weighting: the effect of both attention and confidence should have opposite effects on options whose values are above vs below the reference (Figure 1C). In the following sections, we confirm this and other predictions of the model in two preferential choice experiments. Importantly, these qualitative predictions result from the core Bayesian logic of the model. Thus, while the size of the predicted effects will depend on model parameters (specifically, the cost and precision of each value sample), the qualitative predictions are highly robust (see the Appendix for a formal sensitivity analysis). Moreover, we do not aim to provide a precise algorithmic characterization of the decision-making process, nor a tight quantitative fit to data. Instead, we use the model to illustrate qualitative behavioral signatures of Bayesian updating in value-based choice, specifically the incorporation of prior expectations and uncertainty estimates.

### Considering What We Know

**Figure 2.**
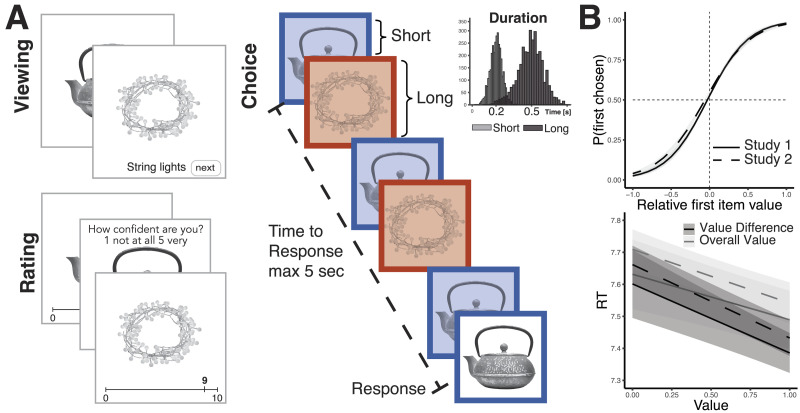
**Paradigm and behavioral results.** (A) In separate phases of the experiment, participants viewed consumer items (twice in Study 1, only), rated them, and (in Study 2, only) indicated the confidence in their ratings, and then chose among sets of two items, each. In the choice phase, items alternated on the screen until a choice was made or 5 s elapsed. Frames around the options color-coded the corresponding response (e.g., left index for blue and right index for red). Presentation durations were manipulated between items, independently of presentation order, response hand, and value, and sampled from long (mean 500 ms) and short (mean 200 ms) duration distributions, respectively. (B) Participants choices in both studies were sensitive to the relative value of options. Participants were faster when value difference was greater (black) and options were more valuable overall (grey). Lines show predictions of fitted linear mixed effects models, error bars show standard errors.

We tested the proposal that people leverage knowledge about the distribution of values in the current choice context to dynamically compensate for uncertainty in two behavioral experiments in which participants made binary choices between consumer items. Most research on attention and choice has used gaze as a proxy for attention, allowing participants to freely fixate between the items in the choice set (Orquin & Mueller Loose, 2013). However, recent research has suggested that this fixation behavior is itself driven by estimated value and uncertainty (Callaway et al., 2021; Jang et al., 2021; Li & Ma, 2021; Song et al., 2019). Thus, to directly measure the causal influence of attention on choice, we instead manipulated fixation time to each item (cf. Armel et al., 2008; Tavares et al., 2017). Specifically, we controlled the order and duration that each item was displayed on the screen (Figure 2A). In both studies, we constructed choice sets to vary in the relative and overall (i.e., average) value of options based on participants’ prior single item ratings that serve as estimates of the true values of the options. In Study 2 participants additionally provided confidence judgments for the initial value ratings (as in Lebreton et al., 2015), which we will examine below.

**Figure 3.**
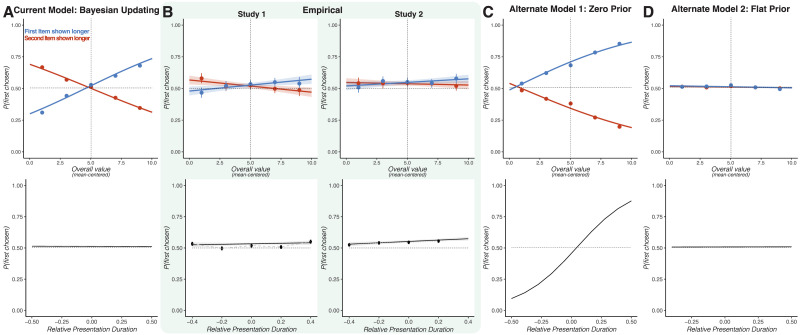
**People rely on contextual information about the expected distribution of values.** (A) According to our Bayesian model, a (relatively) unbiased prior should lead to value-dependent effects of relative presentation on choice, where choice probability increases with relative presentation when the overall (average) value of the options is above the mean, but decreases when overall value is below the mean (top). The model predicts a minimal main effect of relative presentation on choice (bottom). (B) Empirical results in both studies are consistent with the predicted interaction (top, left Study 1, right Study 2) and the corresponding lack of a main effect (left Study 1, right, Study 2). Dots show binned raw data, lines represent linear fits. (C) The flat prior model, with (near) uniform expectations of values does not produce the observed interaction. (D) The zero prior model that overestimates the expected values also does not produce the observed interaction (top) and instead predicts a strong main effect of presentation duration on choice. (A–D) All model predictions were generated with each model’s best fitting parameters; the qualitative trends are robust to parameter settings (see the Parameters section of Methods). Solid lines show predictions of fitted linear mixed effects models, error bars show standard errors.

Our model makes predictions for how choices should deviate from utility maximization based on the true values if participants leveraged expectations about the values of options. As shown in Figure 1C, the momentary value of an item systematically deviates from its true value, depending on the number of samples taken for it and whether its true value is lower or higher than the reference (prior mean). The longer an item is sampled, the more the momentary value should approach the true value. For unconstrained response times, as in the initial single item ratings, we would therefore expect the read-out of the momentary value to be a good proxy for the true value of the items. When choosing among high-value options, more samples should lead to an advantage for that option, whereas when choosing among low-value options, more samples should lead to a disadvantage for that option. We would thus expect that, controlling for the relative value of options, choices would vary as a function of the interaction between the overall value of ones’ options and the relative time spent sampling each of them (Figure 3A). In both studies, the variable of interest is therefore variability in behavior that is not explained by the value ratings. To test whether this residual variability followed the predictions made by our model, we regressed choices onto the relative presentation duration, overall value and their interaction, while controlling for relative value. We will first examine across both studies the predictions for attention effects on behavior and subsequently return to Study 2 to test the predictions for value confidence effects.

Participants’ choices in both studies were sensitive to the values they had assigned in the earlier phase (Supplementary Material Tables S1–S2, Figure 2B). Participants were increasingly likely to choose either item as its value increased relative to the other item, *b*_*S*1_ = 3.76, *t* = 11.12, *p* < .001; *b*_*S*2_ = 3.39, *t* = 11.29, *p* < .001. Participants also made decisions faster when the value difference between items was larger, *b*_*S*1_ = −0.22, *t* = −6.16, *p* < .001; *b*_*S*2_ = −0.23, *t* = −12.83, *p* < .001 and, consistent with previous work (Frömer et al., 2019; Hunt et al., 2012; Smith & Krajbich, 2019), when the overall value of items was higher, *b*_*S*1_ = −0.14, *t* = −4.88, *p* < .001; *b*_*S*2_ = −0.17, *t* = −5.96, *p* < .001. Participants were also sensitive to the presentation order manipulation. In line with previous findings (Krajbich et al., 2010), all else being equal, participants were more likely to choose the first item they had seen, *b*_*S*1_ = 0.10, *t* = 3.80, *p* < .001; *b*_*S*2_ = 0.19, *t* = 5.53, *p* < .001, but this bias decreased for longer response times, *b*_*S*1_ = −0.10, *t* = −3.29, *p* < .001; *b*_*S*2_ = −0.23, *t* = −6.17, *p* < .001.

Controlling for all of these standard effects, we then tested our model’s prediction about the three-way relationship between overall value, relative presentation duration, and choice: People should be more likely to choose items that are presented/seen longer when overall value is high, but less likely to choose them when overall value is low. Confirming this prediction, we found that presentation duration interacted with the overall value of the items, *b*_*S*1_ = 1.75, *t* = 3.80, *p* < .001; *b*_*S*2_ = 1.08, *t* = 2.42, *p* = .016, such that items that were presented longer were more likely chosen when overall value was high, but less likely chosen when overall value was low (Figure 3B).

We can further probe the degree to which participants calibrated their expectations to the distribution of values in their environment by testing the symmetry and strength of the interaction effect. How attention shapes value (and by extension, choice) depends on the mean of the prior (see Figure 1A). When people have calibrated their expectations to the value distribution, i.e., when the prior is unbiased, there should be no net effect of attention on choice. This is because attention has opposite effects on items below vs above the prior mean, and these effects cancel out when the prior mean is the true mean item value. We implement two key alternative models with different priors: The zero prior model assumes a prior of zero (i.e., that each presented item has minimal value), which results in a strictly positive effect of presentation duration on momentary value (for positive valance options, as in our experiment). Such a model has been previously proposed (Jang et al., 2021) and is roughly consistent with the benchmark sequential sampling model of attention effects on choice (attention drift diffusion model [aDDM]; Krajbich et al., 2010, see Callaway et al., 2021 for comparison). The second alternative, the flat prior model, assumes an uninformative prior, so that value estimates do not rely on expectations at all. As shown in Figure 3C and D, both models fail to capture the behavioral pattern: the zero prior model fails to capture the opposite effects of presentation duration for high and low value items, and the flat prior model fails to capture any interaction between overall value and presentation duration. Crucially, this pattern of predictions holds across the full range of plausible parameter values (see the Appendix for a formal sensitivity analysis).

Comparing Figure 3A and B reveals a clear behavioral signature of how well-calibrated our participants’ expectations were. The cross-over point, at which the effect of relative presentation duration on choice is zero, corresponds to the prior mean. In the regression, this cross-over point is captured in the main effect of relative presentation duration on choice (a positive main effect if the cross-over point is below the true mean, and negative if above). Visually, we see that our participants’ cross-over point was just slightly below zero, indicating a mean just slightly below the true empirical mean. Consistent with relatively unbiased prior means in both studies, we found no reliable main effects of presentation duration on choice, *b*_*S*1_ = 0.08, *t* = 0.48, *p* = .634; *b*_*S*2_ = 0.22, *t* = 1.71, *p* = .087 (Figure 3B, bottom). With that said, the prior in Study 2 appeared more biased than in Study 1 as evinced by the larger estimate for the main effect of presentation duration (and the left-shifted cross-over point in Figure 3B, top). Additionally, unlike in Study 1, where the crossover point of the presentation-duration by overall value interaction is quite close to the empirical mean value, in Study 2 the crossover point occurred well below the empirical mean. The model could capture this difference by assuming that participants in study 2 had a more biased prior, specifically one that sits between the unbiased prior and the zero prior and systematically underestimates the value of items (cf. Callaway et al., 2021; Jang et al., 2021).

One plausible explanation for this descriptive difference is that participants in Study 1 had seen all items a second time before rating them and were given the opportunity to exclude unfamiliar items. We omitted this second round in Study 2 to increase the variance in confidence ratings and reduce the overall testing time. These design choices may have also prevented participants from fully updating a global default prior to the experimental context. While it is theoretically possible that participants would refine their prior further throughout the experiment, we find that the rating distributions prior and subsequently to choice in Study 2 are highly similar (cf. Supplementary Material Table S4). Regardless, our overall pattern of results shows that in both studies, participants clearly took information about the contextual distribution of values into account to some extent, and treated very low value items like aversive items (Shenhav et al., 2018; cf. Armel et al., 2008).

Taken together, our results are consistent with a Bayesian updating mechanism that relies on knowledge about the distribution of values in the task. As outlined earlier, this mechanism predicts the same effects for attention and confidence. We tested this prediction using the value confidence ratings in Study 2.

### Considering What We Don’t Know

In Study 2, we tested how confidence in value ratings of the items in isolation affected subsequent choices among pairs of items (see Supplementary Material Table S5 for distributions of value confidence in choice sets, and a replication of the U-shaped relationship with value ratings). Previous work has shown that subjective confidence ratings provide a measure of the precision of one’s value representation and the samples drawn from it (Lebreton et al., 2015; Quandt et al., 2022). Just like attention, confidence—or the lack thereof—should influence the extent to which momentary value estimates are biased towards the prior. Consequently confidence in item values should systematically bias choices between them.

**Figure 4.**
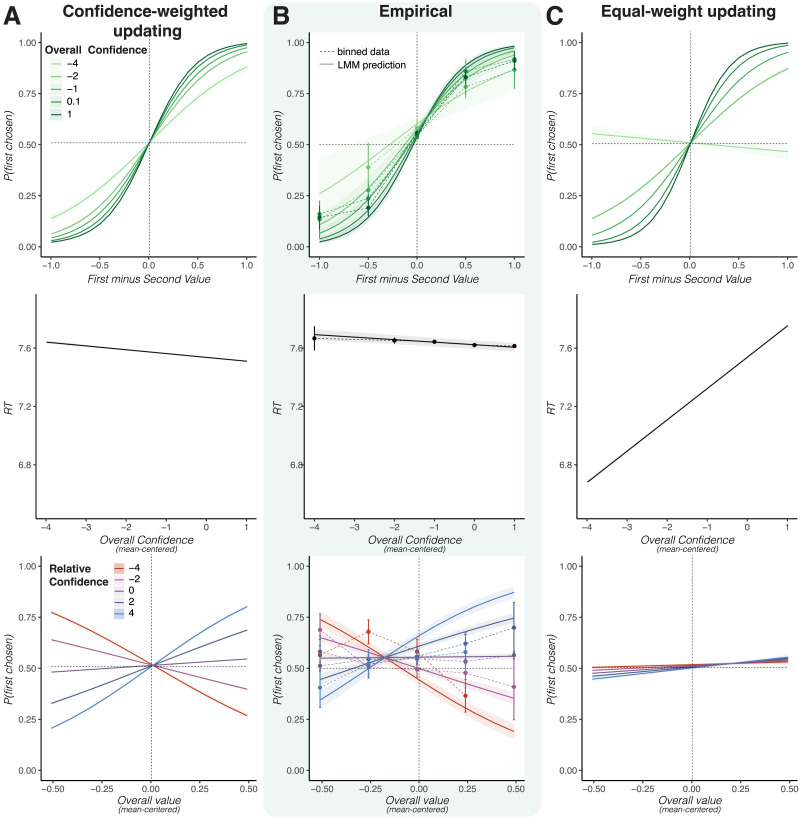
**People update proportional to their confidence in the values of their options**. (A) Our Bayesian updating model predicts more consistent and faster choices when choosing among options with higher overall value confidence (top, center, note: RT is in units of log milliseconds). It also predicts an interaction of relative confidence with value, such that items with higher value confidence than the alternative should be more likely to be chosen when values are above the mean, but less likely when they are below the mean (bottom). (B) Empirical data in Study 2 are consistent with all these predictions. Dots connected by dashed lines show binned raw data residualized for value effects. (C) A model in which the precision of the samples varies with value confidence, but all samples are weighted the same (equal-weight updating) can only capture the increased choice consistency for higher value confidence, but neither of the other effects. It incorrectly predicts slower response times for higher overall confidence (note that we do not show the full range of response times, many of which are far below the range observed in the human data) and no effect of relative confidence. (A–C) Solid lines show predictions of fitted linear mixed effects models, error bars show standard errors. Model predictions were generated with manually selected parameters; the qualitative trends are robust to this choice (see Parameters section of Methods).

A basic prediction of our model is that choices should be more consistent and faster when confidence in both items’ values (overall confidence) is higher (Figure 4A). We can compare these predictions to an alternative equal-weight updating model, in which confidence determines the sample noise but not how the samples are integrated. This alternative model also predicts higher accuracy for higher overall confidence, but at the cost of slower reaction times (see Figure 4C). Consistent with our model’s prediction, Figure 4B shows that when their confidence in both items was higher, participants’ choices were more consistent with their initial ratings (interaction of overall confidence with relative value on choice, *b* = 0.48, *t* = 2.35, *p* = .019) and they responded faster (main effect of overall confidence on RT, *b* = −0.02, *t* = −3.13, *p* = .002, Supplemental Material Table S2). Note, however, that these predictions are not fully robust to the selection of parameters. In particular, there exist parameters for which the main model predicts longer response times with greater confidence, but these parameters also yield lower choice consistency than we observe in the human data (see the Appendix for details).

The key model predictions for relative confidence (confidence in the first item value minus confidence in the second item value) mirror those for relative presentation duration (Figure 4A, bottom; compare Figure 3A) and are not shared by the equal-weight updating model (Figure 4C, bottom). This demonstrates that precision-weighted updating is a crucial component for this prediction. Specifically, the Bayesian updating model predicts that relative confidence will interact with the overall value of options, such that high-confidence items are more likely to be chosen when their values are high and less likely to be chosen when their values are low. Our empirical data confirm this prediction (Figure 4B; interaction between relative confidence and overall value, *b* = 0.63, *t* = 6.59, *p* < .001). Consistent with the biased prior mean mentioned earlier, this effect was not fully symmetric, so that we additionally observed a positive main effect of relative confidence on choice, *b* = 0.11, *t* = 4.32, *p* < .001.

**Figure 5.**
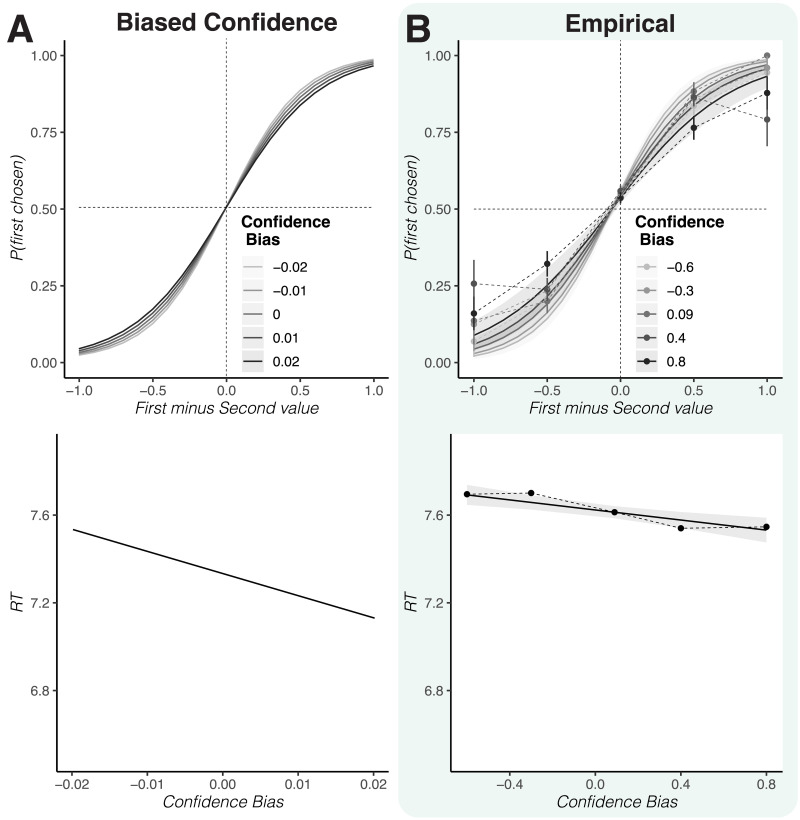
**Overconfidence leads to impulsive choice pattern.** (A) When making the model over- or underconfident, by varying the mapping between value confidence and the precision of the samples, it predicts that increasing overconfidence is associated with decreasing choice consistency and response times. (B) Our empirical results in Study 2 show the same pattern, where people with higher levels of confidence in all value ratings tend to respond less consistently and faster. These results, while consistent with our model are not significant at a conventional threshold. Dots and thin lines show binned raw data residualized for value effects. Lines show predictions of fitted linear mixed effects models, error bars show standard errors.

Our model and task also allow us to explore more subtle deviations from optimal Bayesian metacognition. Specifically, participants may be systematically over- or under-confident, treating samples as if they are more or less precise than they truly are. This would lead over-confident participants to make decisions that are faster and less consistent with their initial ratings, and under-confident participants to make decisions that are slower and more consistent with their initial ratings. As a proxy for overconfidence, we computed the average confidence across all items for each participant. When we include average confidence as a between-subject regressor in our analyses above (Supplemental Material Table S3), we observed the anticipated pattern (Figure 5). Participants with higher confidence tended to be faster, *b* = −0.12, *t* = −1.87, *p* = .060, but less consistent, *b* = −1.13, *t* = −1.74, *p* = .082. We can capture this effect with a biased confidence model that has separate precision parameters for the updating and sampling steps. Although the results do not reach the conventional statistical significance threshold of .05, given that our sample is small and our measure of confidence bias is noisy, these results nevertheless encourage future work better suited to test this mechanism.

## DISCUSSION

When making decisions, we often need to overcome uncertainty due to attention-related delays in information or confidence-related imprecision of that information. In the present study we propose and test a Bayesian-updating account of value-based decision-making as a solution to these challenges. Under this theory, the value one assigns to one’s options depends on two things: 1) one’s expectations about the distribution of values in a given context, and 2) information acquired while considering those options. The relative weight of each component depends on the precision of that information. Crucially, the effect of acquiring more precise information about an option depends on how its true value compares to the expected value. If the true value is higher than expected, more information will tend to increase estimated value; but if the true value is lower than expected, more information will decrease estimated value. This theory thus makes the unique prediction that both increased attention to an option and greater confidence in its value will have opposite effects on choice probability for items that are below vs above the average value of options in the current context. This is exactly what we found.

Our results help shed new light on the role attention plays in decision-making. Previous work has proposed a multiplicative effect of attention and value on choice (Armel et al., 2008; Krajbich et al., 2010; Smith & Krajbich, 2019), such that when attended more, appetitive options would be more likely chosen, whereas aversive items would be less likely chosen. Indeed, only when appetitive and aversive items were included, has a full cross-over been previously demonstrated (Armel et al., 2008). Here we find the same effect, except that in our studies, the “bad” options were not truly aversive, but merely mundane—undesirable only when compared to the alternatives (Shenhav et al., 2018). Our finding that the effect of attention on choice *reverses* for mundane options therefore suggest that people can adapt their reference point, consistent with our Bayesian-updating account, as well as context-dependent range normalization (Khaw et al., 2017) and efficient coding (Polanía et al., 2019) accounts of valuation.

Our task allowed us to disentangle two effects that are typically conflated in studies of the influence of attention on value-based decision-making: the influence of attention on choice and the reverse influence of (beliefs about) choice on attention allocation. One finding that emerged as a result is that, unlike several past studies (Cavanagh et al., 2014; Krajbich et al., 2010; Westbrook et al., 2020), we did not find that attention exerted an independent (additive) influence on the likelihood of choosing one option or another, over and above its role in enhancing the influence of value on choice.

There are at least two potential reasons for this discrepancy. First, participants’ expectations may have been biased in previous studies (Callaway et al., 2021; Jang et al., 2021). With few exceptions, in past studies only options that were positively evaluated during the initial rating or bidding phase were included in the subsequent choice sets. These excluded items may have nevertheless exerted an influence on subsequent expectations about the distribution of option values (cf. Shenhav et al., 2018). This may have produced a prior closer to the mean-zero prior we introduced above, which predicts a main effect of attention on choice. However, this explanation may not be sufficient to explain the additive effect of attention on choice observed in previous studies, given that studies which include stimuli that were associated with negative feedback or mental effort (and were thus clearly aversive) still observed such an additive effect (Cavanagh et al., 2014; Westbrook et al., 2020).

The second potential explanation is that the relationship between attention and choice is not strictly unidirectional (Callaway et al., 2021; Gluth et al., 2018, 2020). In particular, participants in previous studies may have chosen to sample more information about the option they thought was best, producing a correlation between attention and choice through an entirely different causal mechanism (Kaanders et al., 2022; Shimojo et al., 2003). In our study, we intentionally broke the feedback-loop between the decision-process and gaze by experimentally manipulating presentation duration. Along these lines, it is notable that the only study that has shown a full crossover effect of attention and value on choice (Armel et al., 2008) also experimentally manipulated relative attention. In both our study and theirs, disabling active information-search may have eliminated the positive main-effect of gaze on choice. Future work will need to explicitly test this interpretation by comparing free-viewing and experimenter determined gaze in the same task.

Our value confidence results suggest that even when people can’t actively seek information about specific options, they integrate novel information in a controlled way rather than simply uniformly accumulating information. In particular, low-confidence information appears to be down-weighted in the evidence integration. This echoes a model recently proposed by Lee and Usher (2023) in which confidence scales the drift rate of a DDM—a mechanism that they motivate by appeal to Bayesian principles. We build on this work by providing a fully Bayesian model that simultaneously accounts for both value confidence and attention. This allowed us to characterize implications of Bayesian updating beyond simply down-weighting imprecise information, including modulating the influence of expectations (Figure 4) and the possibility of a dissociation between subjective confidence in and objective informativeness of one’s sampled information (Figure 5).

Like attention, value confidence has been proposed to confer a choice benefit at least as long as options are appetitive (Lee & Coricelli, 2020; Li & Ma, 2021). On its face, this proposal resonates with ubiquitous uncertainty aversion, as well as the idea that confidence itself may be a value signal (Lebreton et al., 2015). Here we show that, as with attention, the influence of value confidence on choice critically depends on a decision-maker’s expectations. This finding speaks against uncertainty aversion (or its converse, confidence bonus) as the main driver of choice variability associated with value confidence (Li & Ma, 2021). Rather it suggests a more nuanced role for value confidence in decision-making.

Our choice paradigm isolated one specific role for value confidence: weighing information in belief-updating. To the extent that the decision-maker thinks that the sampled information is precise (i.e., their confidence is high), they will adaptively reduce uncertainty by relying on samples versus expectations. This role parallels similar mechanisms in learning, where learners rely on response-based versus expectation-based predictions when they believe their evaluations are precise (Frömer et al., 2021). Like this previous work, in our model, adaptive updating is driven by subjective confidence rather than the true precision of one’s representations. This important aspect allows us to generate predictions for individual differences and sets our model apart from alternatives in which confidence merely reflects the signal-to-noise ratio (Lee & Usher, 2023). Our simulations and empirical results suggests that the extent to which one updates their beliefs is determined by their subjective *beliefs* about the precision of their samples, rather than merely the objective precision per se (see also Schiffer et al., 2017). When simulating mismatches between properties of the sampling distribution and subjective value confidence in our model, we produced impulsive, inconsistent behavior. We observed the same overall pattern in participants who were more confident overall. These findings add to a growing literature highlighting the importance of (well-calibrated) metacognition for adaptively regulating information-processing (Frömer et al., 2021; Yeung & Summerfield, 2012).

Value confidence likely plays a broader set of roles in decision-making beyond only regulating dynamic belief updating. For instance, previous work has shown that value confidence is used to regulate explore-exploit trade-offs in learning contexts (Boldt et al., 2019). When attention is not experimentally manipulated, but can be freely allocated, we would expect that value confidence similarly provides inputs to (gaze-linked) information search. Importantly, the relationship between value confidence and gaze should then depend on the context (e.g., whether exploration is worthwhile; Wilson et al., 2014). It should also depend on the absolute level of confidence: if confidence is so low that no amount of sampling can increase certainty in the momentary value estimate to a reasonable level, participants should refrain from sampling an option, as the low expected information gain will not justify paying the cost of sampling. Unpacking this complementary role for value confidence in regulating information-processing in decision-making is an important future direction.

By controlling when and for how long items could be sampled through gaze, we eliminated the feedback-loop that has made it difficult to interpret correlations between value, attention, and choice (discussed above). The flipside of this is that we still need to test predictions of models that both allow for free gaze allocation and incorporate confidence-dependent updating. Indeed, confidence could have complex, time-varying effects on attention allocation. Early in the decision, attending to a high-confidence item produces stronger and thus more valuable information. Later in the decision, however, the value of the high-confidence item will have a very precise estimate, making additional attention to it superfluous. By isolating the downstream effects of confidence on choice, we have provided the foundation for understanding the full cyclic process.

Recent work has outlined how goals and momentary beliefs shape information sampling through gaze (Callaway et al., 2021; Gluth et al., 2020; Jang et al., 2021) and explicit information gathering (Hunt et al., 2016). People’s prior beliefs and the type of question they are trying to answer shape the degree to which they integrate information and seek it to begin with, as evident in confirmation bias (Kaanders et al., 2022; Sharot & Garrett, 2016; Talluri et al., 2018), framing effects (Morewedge & Kahneman, 2010), as well as choice goals’ influence on decision making (Frömer et al., 2019; Sepulveda et al., 2020). How these mechanisms shape value-based choices remains to be understood.

Another open question is whether such goal-directed information search is limited to simply which option is considered, or in fact extends to what type of information is considered about a given option. Most sequential sampling models assume that samples are drawn independently from an underlying distribution, but this need not be so. In the context of memory retrieval, during free recall, people are more likely to sequentially sample items that share features (e.g., category) than dissimilar items (Bousfield & Cohen, 1955), perhaps due to spreading of activation that facilitates the retrieval of related items. These findings are consistent with a sequential sampling model in which successive samples are drawn from a Markov chain, and are hence autocorrelated (Zhu et al., 2018). Based on this work, we would expect autocorrelation in value-based sampling as well. For example, when sampling one positive feature about the tea pot in Figure 1 (nice color), one would be more likely to sample other related positive features about it (great shape), rather than a less related feature or a negative one (might drip).

One canonical finding that might be explained by biased memory sampling is the speeding of choices among options that are more rather than less congruent with one’s choice goal (Frömer et al., 2019; Hunt et al., 2012; Sepulveda et al., 2020; Smith & Krajbich, 2019). Mechanistic explanations of the overall value effect on choice include attentional biases (Sepulveda et al., 2020; Smith & Krajbich, 2019), and nonlinear dynamics of the decision-process (Frömer et al., 2019; Hunt et al., 2012). In a reference-dependent value coding scheme as demonstrated here, none of the current models are able to account for the overall value effect on decision time. In fact, our model can only produce it when given a slightly biased prior as observed in the empirical data and then the simulated effects are still much weaker than those found in the data. This tension between predicted and observed findings might suggest that caution is warranted in assuming a role for reference-dependence in this process. However, given the convergent evidence of reference-dependence from our remaining results and other lines of work (Khaw et al., 2017; Polanía et al., 2019; Shenhav et al., 2018), a more plausible alternative is that goal-congruent information is sampled with greater ease, at a faster rate than goal-incongruent information, in line with confirmation and positive evidence biases in information sampling (Hunt et al., 2016; Kaanders et al., 2022; Sharot & Garrett, 2016; Talluri et al., 2018). Future work will need to explicitly manipulate prior expectations and choice goals to arbitrate between different accounts.

Our findings have implications for interpretations of neural correlates of value in value-based choice. Traditionally, neuroeconomics has focused on valuation and evidence accumulation when interpreting neural correlates of value, for instance in dorsal anterior cingulate cortex (Hare et al., 2011; Pisauro et al., 2017). However, these regions have also been associated with goal-directed regulation of information gain (McGuire et al., 2014; O’Reilly et al., 2013), and cognitive control more generally (Shenhav et al., 2013). Our results contribute to a growing consensus that value-based choice draws on those regulatory mechanisms, suggesting that the observed activity in dACC could reflect information regulation rather than accumulated evidence per se (Hunt, 2021; Hunt et al., 2018, 2021; Kaanders et al., 2021; Monosov, 2017).

By characterizing the specific quantities associated with information regulation in the service of decision making (e.g., value confidence, estimate precision, changes in choice certainty), our model can inform specific predictions for neural activity and crucially how it unfolds over time (cf. Frömer et al., 2021; McGuire et al., 2014; O’Reilly et al., 2013). Testing these predictions will allow us to re-evaluate and extend beyond previously identified correlates of evidence accumulation in value-based choice (e.g., Frömer et al., 2024; Pisauro et al., 2017; Polanía et al., 2014).

A central motivation for the decision sciences is to understand the ways in which decision-making deviates from classical rationality and to use that knowledge to improve decision-making. Viewing decision-making as an active process, involving information search and regulation of information-processing shaped by goals and metacognition, provides us with a whole new set of levers to achieve this goal. Perhaps if we understand how these control mechanisms work, we can help decision-makers and learners alike by teaching them to consider and control what they know and what they don’t know.

## METHODS

### Experiment

#### Participants.

Participants were recruited from Brown University and the general community. Thirty participants (19 female) with an average age of 19.6 (*SD* = 1.8) took part in Study 1. Thirty one participants (23 female) with an average age of 21.1 (*SD* = 3.5) took part in Study 2. Participants gave informed consent and were remunerated with 10 USD per hour or course credits. The studies were approved by Brown University’s institutional review board. Sample sizes were determined based on previous studies (Gluth et al., 2018; Krajbich & Rangel, 2011).

#### Task and Procedure.

In Study 1, the experiment consisted of three phases: 1) item familiarization, 2) item rating, and 3) choice. During item familiarization, participants viewed consumer items on the screen individually in a self-paced manner. Items were presented in grey scale and short labels were displayed below the items. Following a first round of viewing all items, participants were presented with each item a second time without labels and asked to indicate items that they could not identify without a label present. These items were removed from subsequent phases of the experiment. In the subsequent rating phase participants were asked to rate how much they liked the remaining items individually on a scale from 0 (not at all) to 10 (a great deal). They were encouraged to use the entire range of values rather than just the extremes. Based on the individual ratings, we constructed personalized choice sets that varied in the relative and overall values of options. Choice sets were created by sorting pairs of products into four conditions based on their rank-ordered subjective rating values: high (target 40 trials), medium (target 40 trials), and low (target 40 trials) value pairs with zero or close to zero value difference, and mixed pairs with value difference covering the entire range (target 120 trials) (Frömer et al., 2019; Shenhav & Buckner, 2014; Shenhav & Karmarkar, 2019; Shenhav et al., 2018). Depending on the range of values participants provided and the number of items they excluded during familiarization, the number of choice sets we could generate varied between 128 and 240 (Median = 228, *M* = 222, *SD* = 24).

In the choice phase, participants viewed pairs of options alternating centrally on the screen inside frames that color coded the corresponding response hands (index fingers placed on A and J keys on a standard keyboard). We manipulated the relative presentation duration of items so that one item was always presented longer than the other. While items alternated on the screen, specific durations on each turn were drawn from different distributions for long (*M* = 500 ms, *SD* = 100 ms) and short presentations (*M* = 200, *SD* = 50); these distributions were informed by previous work (Frömer et al., 2024). Presentation duration, response hand, and order of presentation varied independently. Items alternated until participants made a choice or 5 s had elapsed. After making their choice, participants were presented with the item they chose for one second and allowed to reverse their choice within 750 ms of feedback presentation (5.6% of all trials). Participants performed multiple rounds of practice with empty frames only, letters, and with practice items, to learn the button mapping and get comfortable with the task.

Study 2 differed from Study 1 in three ways: 1) participants performed only the first round of familiarization and no items were excluded, 2) in the rating phase individual item ratings were immediately followed by a prompt to indicate the confidence in that rating, and 3) item ratings were repeated after the choice phase. We removed the second round of familiarization to reduce overall testing time and to include items with very low value confidence. Participants rated their confidence in the value ratings on a scale from 1 (not at all) to 5 (very) by pressing the corresponding number key on a keyboard. The second round of value ratings following the choice phase did not include confidence ratings. Participants performed 240 choice trials unless their rating distributions afforded less (Median = 240, Mean = 236, *SD* = 7). On average they indicated they had made an error on 5.9% of all trials.

#### Analyses.

Our primary interest was participants’ behavior during the choice phase. We analyzed the probability of choosing the first item using generalized mixed effects models with a binomial link function, and RTs using linear mixed effects models using lme4 (Bates, Maechler, et al., 2015) in R (Version 3.6.1). We modeled random intercepts for participants and included random slopes for predictors if supported by the data (Bates, Kliegl, et al., 2015; Matuschek et al., 2017). We performed model selection using the buildmer package. We analyzed choices and RTs of initial choices, including choices that were subsequently reversed after the chosen option was shown. However, choices that were made before the second item was seen were excluded from all analyses (0.7% in Study 1, 0.3% in Study 2). The remaining choices were analyzed with relative value (first item value minus second item value), overall value (average value of both items in a set), relative presentation duration (proportion of time the first item was on the screen), and RT as an index of total decision time. To test how relative presentation duration influences values’ relationship with choice behavior, we modeled interactions between relative presentation duration and value regressors. We analyzed response times with value difference (absolute difference between item values) and overall value. In Study 2, we further included three variables to assess the impact of value confidence on choice: relative confidence (confidence in the first item value minus confidence in the second item value), overall confidence (mean confidence in both items, centered within participants), and confidence bias as a between subject regressor (average confidence of a participant across all items). To test how value confidence impacts values’ relationship with choice behavior, we modeled interactions between confidence and value regressors.

### Model

Here we give further details on the implementation of the model. We focus on the main model based on optimal Bayesian updating, but also highlight the key dimensions that we modify in the alternative models: zero prior, flat prior, equal-weight updating, and biased confidence.

#### Prior.

Following previous work (Callaway et al., 2021), we assume that the prior takes the form of a Gaussian distribution fit to the actual empirical distribution of values, that is:

$$\begin{array}{l} \mu_{\text{prior}}=\text{mean}\left(\text{ratings}\right)\\ \lambda_{\text{prior}}=\text{std}{\left(\text{ratings}\right)}^{-2}. \end{array}$$ (3)

Here, std(ratings) denotes the standard deviation of the value ratings for all items used in the choice phase. Note that, in contrast to Callaway et al. (2021), we assume a perfectly calibrated, or “unbiased” prior, in order to best illustrate the predictions of optimal Bayesian updating.

In the zero prior model, we set *μ*_prior_ to 0. In the flat prior model, we set *λ*_prior_ to 10^−6^, effectively removing all prior information.

#### Confidence.

Value confidence is captured in the precision term, *τ*^(*i*)^. More precisely, *τ*^(*i*)^ is the precision of samples for an item when it is attended. We assume that precision varies by item, and that this variance is related to the rating confidence judgments. For simplicity, we assume that an item’s precision is simply proportional to its confidence rating:

$$\tau^{\left(i\right)}=\beta_{\text{conf}}\cdot \left({\text{conf}}^{\left(i\right)}\right),$$ (4)

where *β*_conf_ is a free parameter (fit to data as described below). Importantly, this equation is just a simple way to generate predictions with minimal additional assumptions; the true relationship between confidence and precision is certainly more complex. For Experiment 1, where we do not have confidence ratings, we use the average confidence rating from Experiment 2 (4.18) for all items.

In the main model, we assume that both the samples themselves and the posterior estimate (given the samples) are constructed using a single *τ*^(*i*)^ parameter, as in optimal Bayesian inference. However, we also consider the possibility that the posterior is formed assuming an incorrect precision, replacing *τ*^(*i*)^ with $\tilde{\tau }$^(*i*)^ in Equation 2. We consider two forms of incorrect precision. In the equal-weight updating model, the agent treats all items as though they have the same sampling precision, specifically the average precision:

$${\tilde{\tau }}^{\left(i\right)}=\beta_{\text{conf}}\cdot \text{mean}\left(\text{conf}\right).$$ (5)

In the biased confidence model, the agent assumes that sampling precision is systematically higher or lower than it really is:

$${\tilde{\tau }}^{\left(i\right)}=\beta_{\text{bias}}+\beta_{\text{conf}}\cdot {\text{conf}}^{\left(i\right)},$$ (6)

where *β*_bias_ > 0 corresponds to overconfidence.

Critically, in both of these models, the true precision of the samples (Equation 1) remains unchanged. It is only the posterior estimate (Equation 2) that uses the biased $\tilde{\tau }$^(*i*)^ term. That is, the distribution from which samples are drawn is the same as in the default model; it is only the noise that the model assumes when it weighs the information that is different. Differences in performance are thus specific to confidence-weighted updating and not driven by differences in sampling noise.

#### Response Times and Decision Rule.

The model’s response time for a given trial is proportional to the number of samples taken before a choice is made (we arbitrarily assume 10 ms per sample; see below). The maximum number of samples within the response deadline of 5 s is thus 500. As in the human data, any decisions not made within this time are aborted and excluded from further analysis. Intuitively, one should stop sampling (and make a decision) when the cost of additional samples exceeds the expected increase in decision quality from collecting those samples. We assume the cost is linear with time (which is proportional to the number of samples). The cost-per-second is a free parameter.

The number of samples is determined by the model’s “stopping rule”. Previous work has used dynamic programming to identify the optimal stopping rule (Drugowitsch et al., 2012; Tajima et al., 2016). However, the exogenous attention manipulation complicates this approach, and the exact shape of the stopping rule is not of critical interest for the current study. Thus, we instead employ a simple approximation to the optimal stopping rule. The approximation has two parts: First, rather than predicting the specific sequence of future presentation durations, we assume that all future samples will be split between the items according to the ratio of their average presentation durations. Second, we estimate the value of additional sampling using an adaptation of the Directed Cognition model proposed by Gabaix and Labison (2005). Concretely, we compute the expected increase in decision quality (the value of the chosen option) for different amounts of additional sampling, and substract the cost of that additional sampling. If there is any amount of sampling for which this value is positive, the model continues sampling. See the Appendix for further details.

The decision rule simply selects whichever item has maximal estimated value when sampling terminates. Assuming this happens at time *T*, the choice is thus:

$$\text{choice}=\underset{i}{\text{argmax}}\;u_{T}^{\left(i\right)}$$ (7)

note that these stopping and decision rules could, in principle, be implemented as a pair of time-varying thresholds that depend on the trial-specific confidence ratings and average presentation durations (cf. Jang et al., 2021).

#### Parameters.

The model has two free parameters: *β*_conf_ specifies how sampling precision scales with confidence and *c* species the sampling cost. These parameters play a similar role to the drift rate and threshold parameters in the DDM. Implicitly, there is an additional parameter, *δt*, which specifies the time per sample. However, this parameter simply controls the temporal precision of the predictions; we set it to *δt* = 10 ms.

The other two parameters of the model were fit to human data. In principle, these parameters could be fit by maximum likelihood estimation at the trial level. However, we found that the considered models were unable to capture the trial-by-trial RT distribution, leading to degenerate parameter estimates that predicted essentially random behavior. Given that a very similar model was successfully fit in a free-viewing experiment (Callaway et al., 2021), we conjecture that the quantitative mismatch stems from differences in how people and the model react to the external manipulation of attention.

Fortunately, the key predictions of our model are only weakly sensitive to the specific choice of parameters. We thus fit the parameters at the group level by minimizing mean squared error over a minimal set of summary statistics: accuracy (proportion of correct choices, excluding trials in which items were of equal value) and median RT. We estimated these statistics for each candidate parameter configuration by their empirical value in a simulated dataset (with 30 repetitions of each human trial to reduce variance). We put accuracy and RT on a common scale by dividing them by their theoretical maximum values (1 for accuracy and 5 s for RT). The total loss was then defined as the sum of the squared difference between the model-predicted and human summary statistics. We minimized this loss with a grid search, considering 30 values for each parameter in the range (0.001, 0.1), spaced logarithmically. Parameters were fit separately for each study-model combination with the exception of the biased confidence model, for which we used the same parameters as the main model to isolate the effect of the confidence bias. All models were able to closely match the observed accuracy (absolute error < 0.7%) and median RT (absolute error < 17 ms). The identified parameters are shown in Table A1.

A critical concern is that the qualitative model predictions may be sensitive to the choice of parameters. In particular, it could be that the proposed model only occasionally produced the behavior that we interpreted as core qualitative predictions. Alternatively, the alternative models could capture the observed behavior with different parameters. Either possibility would undermine our conclusions. To rule out these possibilities, we conducted a sensitivity analysis over the full range of parameters specified above. We found that the proposed model makes the same qualitative predictions in all cases (with the exception of faster response time for higher confidence, as previously discussed). In contrast, the alternative models never capture the observed effects (with the exception of the effect of overall confidence on choice consistency, also discussed above). See the Appendix for details.

## ACKNOWLEDGMENTS

We are grateful to Akari Izumi, Maisy Tarlow, Gloria Feng, and Selin Baydar for assistance in data collection.

## FUNDING INFORMATION

This work was funded by NIH grant 1R01MH124849-01, NIH grant 2P50MH106435, NIH Shared Instrument grant S10 OD025181, and NSF grant 2309022 (AS), and an AMS Springboard award (RF).

## DATA AND CODE AVAILABILITY

All code and data supporting this submission can be found at https://github.com/fredcallaway/BASS.

## Supplementary Material



## Figures and Tables

**Figure A1.**
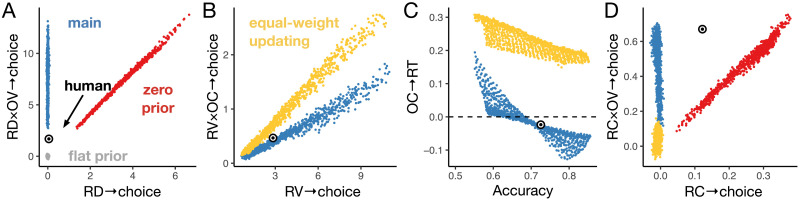
**Sensitivity analysis. Each panel shows the distribution of regression coefficients for each model under a wide range of parameters.** (A) Effects of relative presentation duration (RD) and interaction with overall value (OV) on choice. (B) Effects of relative value (RV) and interaction with overall confidence (OC) on choice. (C) Effect of overall confidence (OC) on response time (RT) as a function of overall choice accuracy. (D) Effects of relative confidence (RC) and interaction with overall value (OV) on choice.

## APPENDIX

### Sensitivity Analysis

To investigate the robustness of the model predictions, we conducted a sensitivity analysis in which we varied the two free parameters (*β*_conf_ and *c*) across a large space and checked for the key effects that we emphasize in the paper. We used the same parameter range and simulations as used for fitting: 30 values for each parameter in the range (0.001, 0.1) on a log scale. Note that the fitted parameters are roughly in the center of this scale; thus, we consider parameters that are roughly an order of magnitude smaller and larger than those used for the main results. We exclude simulated datasets with less than 55% choice accuracy (vs 73% in the human data); accuracy is defined as the proportion of trials with non-zero relative value in which the higher valued item was chosen. We then conducted the choice and response time regressions reported in the main text (without random effects because the model is fit at the group level). We use these regression coefficients to check which models are capable of reproducing the effects plotted in the main text.

#### Value-Dependent Effect Presentation Duration On Choice.

As illustrated in Figure 3 (top), Bayesian updating predicts (1) an interaction between relative presentation duration and overall value, and (2) no main effect of relative presentation duration. Figure A1A plots the distribution of these two coefficients on the y and x axes, respectively. We see that the main model never predicts a main effect of relative presentation duration, and predicts an interaction of widely varying strength (although always stronger than that observed in the human data). In contrast, the zero prior model predicts a strong main effect that scales linearly with the interaction effect, while the flat prior model predicts neither effect.

#### Effect of Confidence On Choice Consistency.

As illustrated in Figure 4 (top), both the main model and the equal-weight model predict a positive interaction between overall confidence and relative value in predicting choice. The distribution of coefficients for relative value and the interaction are shown in Figure A1B.

#### Effect of Confidence On Response Time.

As illustrated in Figure 4 (middle), people respond more quickly when their overall confidence is higher. As shown in Figure A1C, the equal-weight updating model instead predicts longer response times with higher confidence. Interestingly, the main model can also predict a positive effect of confidence on response times, specifically when *β*_conf_ is so low as to make sampling not worthwhile for low confidence items. However, this also yields lower choice accuracy than observed in the human data.

#### Value-Dependent Effect of Confidence On Choice.

As illustrated in Figure 4 (bottom), Bayesian updating predicts (1) an interaction between relative confidence and overall value, and (2) no main effect of relative confidence. In this study, however, participants clearly did show a main effect of relative confidence, as indicated by the leftward shift of the crossover point. Such a prediction would arise from a model in which the prior mean is between zero and the true empirical mean (as used in Callaway et al., 2021). As shown in Figure A1D, the coefficients for the human data lie in between the main model and the zero prior model, indicating that this data would be well captured by a model whose prior is biased towards zero.

### Stopping Rule

As briefly described in the main text, we use a two-part approximation to the optimal stopping rule. Our method approximates the information content of future samples by assuming that both items will be presented in proportion to their average presentation times; it then estimates the value of additional sampling by hypothetically committing to different amounts of sampling. We provide further details on each component below.

#### Approximating Future Presentation Durations.

A truly optimal stopping policy would take into account the specific sequence of future presentations, in particular, using the elapsed time in the current presentation to predict the switch and therefore the precision of upcoming samples. Rather than model this complex (and perhaps psychologically implausible) process, we instead use the proportion of presentation time on each item. That is, we use a mean-field approximation with respect to time. Concretely, we approximate the effect of attention on future value samples for each item as

$${\bar{p}}^{\left(1\right)}=\frac{{\bar{t}}_{1}}{{\bar{t}}_{1}+{\bar{t}}_{2}},$$ (8)

where $\bar{t}$_1_ and $\bar{t}$_2_ are the means of the presentation duration distributions for the two items (either 200 ms and 500 ms or 500 ms and 200 ms). $\bar{p}$^(2)^ is defined analogously.

#### Value of Additional Sampling.

How should one decide whether to make a decision or keep sampling? We approximate the optimal stopping rule using an adaptation of the Directed Cognition model proposed by Gabaix and Laibson (2005). The intuition for the approximation comes in three steps.

First, we can quantify the *value of information* (VOI) for taking one additional sample as the expected increase in the utility of the item that would be chosen with additional information vs immediately:

$${\text{VOI}}_{t}\left(1\right)=\text{E}\left[u_{{\text{choice}}_{t+1}}-u_{{\text{choice}}_{t}}\right].$$ (9)

where *u*_choice_*t*__ is the highest posterior mean value in the choice set at time *t*. We derive a closed-form expression for the VOI below.

Second, if the expected increase in decision quality for taking one more sample is greater than the sample cost, then clearly one should take (at least) one more sample. That is, one should take another sample if

$${\text{VOI}}_{t}\left(1\right)<c$$ (10)

where *c* is the cost per sample, a free parameter of the model.

Third, note that VOI_*t*_(1) underestimates the true value of sampling. This is because it assumes that one has to make a decision after taking one additional sample when one could in fact take more samples. In some cases, it may require multiple samples to change one’s mind; in these cases, sampling once has minimal value, while sampling several times could have substantial value. To mitigate this problem, we can hypothetically commit to taking *N* samples. If the VOI for *N* more samples is greater than the cost of those samples (for any *N*), then it would be better to commit to taking *N* more samples than to make a decision now. However, actually committing to taking every sample would be unwise, as the next sample could make the correct decision obvious, rendering the following *N* −1 superfluous. Thus, we recompute this value at each time step, stopping as soon as

$$\max_{N}{\text{VOI}}_{t}\left(N\right)<Nc$$ (11)

#### Derivation of the VOI.

Finally, we derive a closed-form expression for Equation 9 for arbitrary *N*. First, note that

$$\text{E}\left[u_{{\text{choice}}_{t}}\right]=\max\left\{\mu_{t}^{\left(1\right)},\mu_{t}^{\left(2\right)}\right\}$$ (12)

because one chooses the item with higher expected value, $\mu_{t}^{\left(i\right)}$, and the expected value of that item is simply $\mu_{t}^{\left(i\right)}$. Thus we can rewrite Equation 9 as

$${\text{VOI}}_{t}\left(1\right)=\text{E}\left[\max\left\{\mu_{t+1}^{\left(1\right)},\mu_{t+1}^{\left(2\right)}\right\}\right]-\max\left\{\mu_{t}^{\left(1\right)},\mu_{t}^{\left(2\right)}\right\}.$$ (13)

Here, $\mu_{t+1}^{\left(1\right)}$ and $\mu_{t+1}^{\left(2\right)}$ are random variables describing the posterior means of each item after one more sample is collected. $\mu_{t+1}^{\left(i\right)}$ is normally distributed with mean $\mu_{t}^{\left(i\right)}$ and standard deviation

$$\zeta_{N}^{\left(i\right)}=\frac{N{\tilde{\tau }}^{\left(i\right)}{\bar{p}}^{\left(i\right)}}{\left(\lambda_{t}+N{\tilde{\tau }}^{\left(i\right)}{\bar{p}}^{\left(i\right)}\right)}\sqrt{\frac{1}{\lambda_{t}}+\frac{1}{N{\tilde{\tau }}^{\left(i\right)}{\bar{p}}^{\left(i\right)}}}.$$ (14)

where the fraction captures the weight of the sample in the posterior update and the square root term is the standard deviation of the sample given the current posterior mean, taking into account uncertainty in the true value as well as the sampling noise. Finally, using a standard formula for the expectation of the maximum of two Gaussians (Nadarajah & Kotz, 2008), we have

$$\text{E}\left[\max\left\{\mu_{t+N}^{\left(1\right)},\mu_{t+N}^{\left(2\right)}\right\}\right]=\mu_{t}^{\left(1\right)}\Phi \left(\beta \right)+\mu_{t}^{\left(2\right)}\Phi \left(-\beta \right)+\phi \left(\beta \right)$$ (15)

where

$$\beta =\frac{\mu^{\left(1\right)}-\mu^{\left(2\right)}}{\sqrt{{\left(\zeta_{N}^{\left(1\right)}\right)}^{2}+{\left(\zeta_{N}^{\left(2\right)}\right)}^{2}}.}$$ (16)

**Table A1.** Fitted parameters for each model.

Study	Model	*c*	*β*_conf_
1	bayesian updating	0.020	0.013
1	flat prior	0.028	0.015
1	zero prior	0.013	0.017
2	bayesian updating	0.017	0.015
2	equal-weight updating	0.015	0.015
