# Supplementary material for: Considering What We Know and What We Don’t Know: Expectations and Confidence Guide Value Integration in Value-Based Decision-Making
Source: Open Mind (Camb). 2025 Jun 25;9:791–813. doi: 10.1162/opmi.a.3 (PMC12240722; doi:10.1162/opmi.a.3)
Supplement: Supplementary file 1 [file opmi-09-791-s001.docx]

**Supplementary Information**

Study 1

Table S1. Choice behavior as a function of Value and Attention

|  | **P(First chosen)** | | | | **log RT** | | | |
| --- | --- | --- | --- | --- | --- | --- | --- | --- |
| *Predictors* | *Log-Odds* | *CI* | *t* | *p* | *b* | *CI* | *t* | *p* |
| (Intercept) | 0.10 | 0.05 – 0.16 | 3.80 | **<0.001** | 7.56 | 7.47 – 7.66 | 153.65 | **<0.001** |
| First vs second item value | 3.76 | 3.10 – 4.42 | 11.12 | **<0.001** |  |  |  |  |
| Relative first item presentation duration (RPD) | 0.08 | -0.26 –   0.42 | 0.48 | 0.634 |  |  |  |  |
| RT | -0.10 | -0.17 – -0.04 | -3.29 | **0.001** |  |  |  |  |
| Overall Value (OV) | -0.09 | -0.28 – 0.11 | -0.89 | 0.374 | -0.14 | -0.20 –  -0.08 | -4.88 | **<0.001** |
| RPD by OV | 1.76 | 0.85 – 2.67 | 3.80 | **<0.001** |  |  |  |  |
| Value Difference |  |  |  |  | -0.22 | -0.28 –  -0.15 | -6.16 | **<0.001** |
| **Random Effects** | | | | | | | | |
| σ^2^ | 3.29 | | | | 0.11 | | | |
| τ_00_ |  | | | | 0.07 _Participant_ | | | |
| τ_11_ | 2.77 _First vs SecondValue_ | | | | 0.03 _Value Difference_ | | | |
|  | 0.44 _RPD_ | | | | 0.02 _Overall Value_ | | | |
| ρ_01_ |  | | | | -0.91 | | | |
|  |  | | | | -0.42 | | | |
| ICC | 0.08 | | | | 0.41 | | | |
| N | 30 | | | | 30 | | | |
| Observations | 6563 | | | | 6612 | | | |
| Marginal R^2^ / Conditional R^2^ | 0.266 / 0.322 | | | | 0.022 / 0.423 | | | |

Is First Item Chosen~ Relative Value (first -second)+ RPD+ RT+ OV+ RPD:OV+ (0 + Relative Value (first -second)+ Relative First Item Presentation Duration| Participant);

log(RT*1000)~ Value Difference + OV + (Value Difference + OV| Participant)

Study 2

Table S2. Choice behavior as a function of Attention, Values and Value Confidence

|  | **P(First chosen)** | | | | **log RT** | | | |
| --- | --- | --- | --- | --- | --- | --- | --- | --- |
| *Predictors* | *Log-Odds* | *CI* | *t* | *p* | *b* | *CI* | *t* | *p* |
| (Intercept) | 0.19 | 0.12 – 0.26 | 5.53 | **<0.001** | 7.62 | 7.57 – 7.68 | 261.36 | **<0.001** |
| First vs second item value | 3.39 | 2.80 – 3.98 | 11.29 | **<0.001** |  |  |  |  |
| RT | -0.23 | -0.30 –  -0.15 | -6.17 | **<0.001** |  |  |  |  |
| Relative Confidence | 0.11 | 0.06 – 0.16 | 4.32 | **<0.001** | -0.01 | -0.02 –  -0.01 | -3.72 | **<0.001** |
| Relative first item presentation duration (RPD) | 0.22 | -0.03 –  0.47 | 1.71 | 0.087 |  |  |  |  |
| Overall Confidence | -0.05 | -0.13 –   0.03 | -1.30 | 0.193 | -0.02 | -0.03 –  -0.01 | -3.13 | **0.002** |
| Overall Value (OV) | -0.01 | -0.20 –   0.18 | -0.06 | 0.954 | -0.17 | -0.22 –  -0.11 | -5.96 | **<0.001** |
| First vs second item value by Overall Confidence | 0.48 | 0.08 – 0.89 | 2.35 | **0.019** |  |  |  |  |
| Relative Confidence by OV | 0.63 | 0.44 – 0.82 | 6.59 | **<0.001** |  |  |  |  |
| RPD by OV | 1.08 | 0.21 – 1.96 | 2.42 | **0.016** |  |  |  |  |
| Value Difference |  |  |  |  | -0.23 | -0.26 –  -0.19 | -12.83 | **<0.001** |
| **Random Effects** | | | | | | | | |
| σ^2^ | 3.29 | | | | 0.08 | | | |
| τ_00_ | 0.02 _Participant_ | | | | 0.03 _Participant_ | | | |
| τ_11_ | 2.18 _Participant.Relative Value_ | | | | 0.02 _Participant.Overall Value_ | | | |
|  |  | | | | 0.00 _Participant.Value Difference_ | | | |
| ρ_01_ | 0.25 _Participant_ | | | | 0.07 | | | |
|  |  | | | | -0.48 | | | |
| ICC | 0.06 | | | | 0.25 | | | |
| N | 31 | | | | 31 | | | |
| Observations | 7259 | | | | 7259 | | | |
| Marginal R^2^ / Conditional R^2^ | 0.249 / 0.293 | | | | 0.048 / 0.288 | | | |

Is First Item Chosen ~ Relative Value (first -second)+ RT+ Relative Confidence+ RPD+ Overall Confidence+ Relative Value :Overall Confidence+ OV+ Relative Confidence:OV+ RPD:OV+ (1 + Relative Value (first -second)| Participant);

log(RT*1000) ~ Value Difference + OV+ Relative Confidence+ Overall Confidence+(OV+Value Difference|Participant)

Table S3. Choice behavior as a function of Attention, Values, Value Confidence and Confidence Bias

|  | **P(First chosen)** | | | | **log RT** | | | |
| --- | --- | --- | --- | --- | --- | --- | --- | --- |
| *Predictors* | *Log-Odds* | *CI* | *t* | *p* | *b* | *CI* | *t* | *p* |
| (Intercept) | 0.19 | 0.13 – 0.26 | 5.59 | **<0.001** | 7.62 | 7.57 – 7.68 | 282.25 | **<0.001** |
| First vs second item value | 3.38 | 2.82 – 3.94 | 11.91 | **<0.001** |  |  |  |  |
| RT | -0.23 | -0.30 –  -0.16 | -6.20 | **<0.001** |  |  |  |  |
| Relative Confidence | 0.11 | 0.06 – 0.16 | 4.31 | **<0.001** | -0.01 | -0.02 –  -0.01 | -3.73 | **<0.001** |
| Relative first item presentation duration (RPD) | 0.22 | -0.03 –  0.47 | 1.71 | 0.088 |  |  |  |  |
| Overall Confidence | -0.05 | -0.13 –  0.03 | -1.31 | 0.190 | -0.02 | -0.03 –  -0.01 | -3.13 | **0.002** |
| Confidence Bias | -0.06 | -0.22 –  0.09 | -0.80 | 0.423 | -0.11 | -0.24 –   0.01 | -1.87 | 0.062 |
| Overall Value (OV) | -0.01 | -0.20 –   0.18 | -0.06 | 0.951 | -0.17 | -0.22 –  -0.11 | -5.97 | **<0.001** |
| First vs second item value by Overall Confidence | 0.48 | 0.08 – 0.89 | 2.33 | **0.020** |  |  |  |  |
| First vs second item value by Confidence Bias | -1.13 | -2.40 –   0.14 | -1.74 | 0.082 |  |  |  |  |
| Relative Confidence by OV | 0.63 | 0.44 – 0.82 | 6.57 | **<0.001** |  |  |  |  |
| RPD by OV | 1.08 | 0.21 – 1.96 | 2.42 | **0.016** |  |  |  |  |
| Value Difference |  |  |  |  | -0.23 | -0.26 –  -0.19 | -12.65 | **<0.001** |
| **Random Effects** | | | | | | | | |
| σ^2^ | 3.29 | | | | 0.08 | | | |
| τ_00_ | 0.02 _Participant_ | | | | 0.02 _Participant_ | | | |
| τ_11_ | 1.89 _Participant.Relative Value_ | | | | 0.02 _Participant.Overall Value_ | | | |
|  |  | | | | 0.00 _Participant.Value Difference_ | | | |
| ρ_01_ | 0.19 _Participant_ | | | | 0.10 | | | |
|  |  | | | | -0.31 | | | |
| ICC | 0.05 | | | | 0.23 | | | |
| N | 31 | | | | 31 | | | |
| Observations | 7259 | | | | 7259 | | | |
| Marginal R^2^ / Conditional R^2^ | 0.250 / 0.288 | | | | 0.072 / 0.281 | | | |

Is First Item Chosen ~ Relative Value (first -second)+ RT+ Relative Confidence+ RPD+ Overall Confidence+ Relative Value :Overall Confidence+ Confidence Bias+ OV+ Relative Value :Confidence Bias+ Relative Confidence:OV+ RPD:OV+(Relative Value (first -second)| Participant); log(RT*1000) ~ Value Difference + OV+ Relative Confidence+ Overall Confidence +Confidence Bias+(OV+Value Difference|Participant)

**Biased prior**

In our main model, we assumed a prior that was perfectly calibrated to the distribution of items in the experiment. However, previous work (Callaway et al. 2021) found that participant behavior could be better captured by assuming a slight negative bias in this prior, that is, assuming that people expect items to be worse than they are on average. Following Callaway et al., we model this by downscaling the prior mean by a free parameter, α. Including this parameter allows us to capture the small, non-significant main effect of attention on choice (see Supplementary Figure 2A) and the effect of overall value on response time. As shown in Supplementary Figure 2 B and C, this can be seen in the offset of the crossover point at which relative fixation time or relative confidence have no impact on choice.


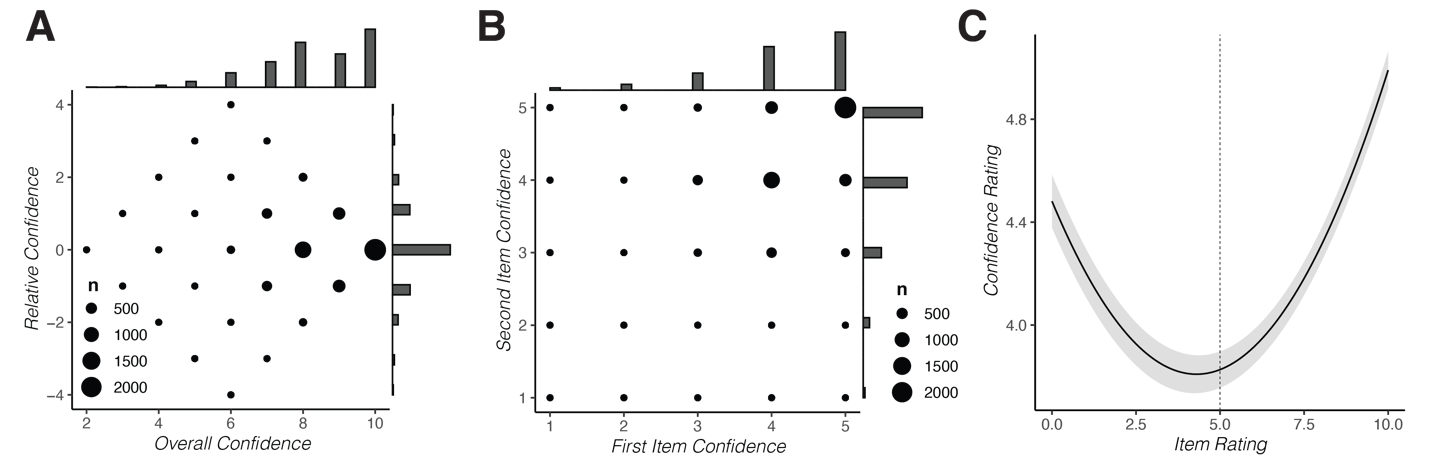


**Supplementary Figure 1.** **Confidence metrics.** **A.** Joint and marginal distributions of Overall and Relative Confidence. **B.** Joint and marginal distributions of First Item Value Confidence and Second Item Value Confidence. **A.-B.** Joint distributions are shown as size-coded dots, marginal distributions are shown as histograms. **C.** Relationship between Value Confidence and Item Rating. The U-shape pattern replicates previous findings and shows that Item Rating and Value Confidence vary independently.

Table S4

|  | **Rating 1** | **Rating 2** | **Rating Deviation** |
| --- | --- | --- | --- |
| Mean | 5.11 | 5.06 | 1.38 |
| SD | 3.09 | 2.91 | 1.4 |

Note: Ratings are correlated r = 0.79, p < .001

Table S5. Ratings made with higher Confidence are more consistent, so are higher ratings and more extreme ratings.

|  | **Rate Deviation** | | | |
| --- | --- | --- | --- | --- |
| *Predictors* | *b* | *CI* | *t* | *p* |
| (Intercept) | 1.31 | 1.10 – 1.52 | 12.03 | **<0.001** |
| Confidence | -0.14 | -0.20 – -0.09 | -5.42 | **<0.001** |
| Rating1 | 0.13 | 0.08 – 0.18 | 4.99 | **<0.001** |
| Rating1 * Rating1 | -0.02 | -0.02 – -0.01 | -8.37 | **<0.001** |
| **Random Effects** | | | | |
| σ^2^ | 1.71 | | | |
| τ_00_ _Participant_ | 0.32 | | | |
| τ_11_ _Confidence_ | 0.01 | | | |
| τ_11_  _Rating1_ | 0.01 | | | |
| ρ_01_ | 0.45 | | | |
|  | -0.74 | | | |
| ICC | 0.13 | | | |
| N _Participant_ | 31 | | | |
| Observations | 10184 | | | |
| Marginal R^2^ / Conditional R^2^ | 0.030 / 0.152 | | | |
